# Polymeric Interlayer Strengthening with Boron Neutron Capture Radiation Treatment for Laminated Glass

**DOI:** 10.3390/polym15071672

**Published:** 2023-03-28

**Authors:** Joseph C. Philipps, John M. Gahl, Hani A. Salim, John D. Brockman, Michael C. Newberry

**Affiliations:** 1 Department of Civil and Environmental Engineering, University of Missouri, Columbia, MO 65211, USA; 2 Department of Electrical Engineering and Computer Science, MU Research Reactor, University of Missouri, Columbia, MO 65211, USA; 3 Department of Chemistry, University of Missouri, Columbia, MO 65211, USA; 4 Battelle at Tyndall, Air Force Civil Engineer Center, Tyndall Air Force Base, FL 32403, USA

**Keywords:** laminated glass, polymer interlayer, hardening, boron neutron capture treatment, polymer crosslinking, high strain rate

## Abstract

Polymer interlayer materials are utilized in laminated glass systems to provide increased resilience from blast incidents. The polymer chains within the interlayer material can benefit from material modifications that increase the crosslinking between adjacent chains. One theorized method of targeted crosslinking is made possible through a boron neutron capture process. This process utilizes neutron radiation that bombards boron material, thus producing emissions of highly energetic particles into the polymer. The method has been experimentally utilized for bulk material processing as well as surface treatment. The surface treatment process has been extensively investigated in this study to manipulate polymers commonly used as interlayer material. Comparison evaluation tests have been completed to show the material behavior change through static tensile loading, dynamic tensile loading, indentation testing, and scratch resistance testing. Results present the specific material behavior changes, effects on different interlayer material, and optimizations for the treatment processes. Data resulting from these tests will expand the understanding of the material behavior changes from treatment techniques and show evidence of the expected crosslinking. This understanding will lead to a quantifiable application of system capacities to improve the future designs of the window and building systems and lead to a safer, more secure, and resilient infrastructure. Polymer treatment by boron neutron capture radiation has produced polymer interlayers with the potential of increased resilience to blast. The research to date has evaluated treated polymers and shown that the hardening and increased elasticity of the material can be initiated through treatment, thus indicating crosslinking behavior. These results show distinct changes in the material behavior, particularly with the EVA interlayer material. The harder surface of the interlayer may resist the cutting of the interlayer surface by glass shards. Scratch resistance was 30% higher and the measured hardness was 100% on treated surfaces. Treated EVA exhibited a 40% higher stress capacity, a 35% higher toughness, and a 40% increase in the elasticity of the material. The overall toughness increase of the treated polymer material allows for a higher energy absorption, and an overall improvement of window performance in blast conditions. The treatment technique can be applied to a variety of window interlayer products for optimal material performance in blast conditions.

## 1. Introduction

A critical infrastructure that is either vulnerable to attack or that is located within a high threat area will require increased protection. Increased efforts to strengthen the building structure, particularly with regards to the explosion-resistant window and building envelope systems, can improve the life-safety and continued operability of the critical infrastructure under adverse circumstances. The exterior envelope is the most vulnerable component of a building to an exterior hazard because it is the part of the building closest to the source of the hazard. Among all the exterior envelope components, light elements such as the glass windows are critical points of vulnerability. Improving the performance of laminated glass (LG) panels under severe loads such as a blast depends largely on the characteristics of the glass and the polymer interlayer used. For improved energy absorption and composite integrity of the LG system, the interlayer material performance and surface characteristics are critical for the survivability of the LG system under extreme loading events.

Polymer treatment by boron neutron capture radiation has produced polymer interlayers with the potential of an increased resilience to blasts. The research to date has evaluated treated polymers and shown that the hardening and increased elasticity of the material can be initiated through treatment, thus indicating crosslinking behavior. These results show distinct changes in the material behavior, particularly with the EVA interlayer material. The harder surface of the interlayer may resist the cutting of the interlayer surface by glass shards. The overall toughness of the polymer material is also increased, allowing for a higher energy absorption, and the overall improvement of window performance in blast conditions. The treatment technique can be applied to a variety of window interlayer products for optimal material performance in blast conditions.

To understand a useful link between laminated glass windows and the radiation treatment of polymers, some additional background on these subjects is provided, including a description of laminated glass panels and their behavior under blast loads, the radiation process utilized in the work, the interlayer material types that were evaluated, and the material characterization methods.

### 1.1. Laminated Glass Window Systems

Window systems have advanced to utilize a layered combination of glass and polymer interlayer materials to protect against external hazards, including blast hazards [1,2,3]. The polymer interlayer material itself can vary in its composition, and its performance can be enhanced by variations in the material strength [4]. Methods to manipulate the polymer material can help lead to increased strength and an overall resilience of the window system.

A laminated glass panel consists of two or more layers of glass with a polymeric material between each glass layer. The advantages of this polymeric interlayer are to hold the fragments of the glass when the glass cracks due to blast loads. The cracked laminated glass panel works as a continuous membrane attached to the supporting frame and dissipates a great amount of cracking energy, while preventing injury from projectile glass shards [5,6,7].

### 1.2. Enhancement of Polymeric Interlayer Mechanical Properties by Radiation

Blast protection systems traditionally employ the use of polymers to increase the resilience of glass window systems. These polymers can introduce complementary properties to a traditional system through their unique material characteristics. Past research has shown that the material characteristics of polymers can be manipulated through irradiation [8,9,10,11]. Furthermore, the use of specific material doping, such as the utilization of 10Boron, can create conditions that enable the crosslinking of polymer chains that are exposed to a neutron field and thus harden the polymer. Unlike gamma or beta radiation which treats a bulk material, this technique hardens the material only in the very near proximity of the boron doping. Surfaces or layers internal to the bulk can be preferentially hardened. Similar to light ion hardening of surfaces, the particle flux is correlated with the hardening and cross-linking, but not limited to surfaces [9,11].

Traditional interlayer materials are composed of singular, homogenous polymer types that share their singular resistance behavior with the overall system. The addition of multiple different polymer layers can increase the overall blast resistance of a window system, but it can create concerns with clarity, adhesion, weight, and plasticizer migration. The ability to employ targeted hardening within the depths of a singular, homogenous polymer could increase the variation in resistance behaviors found within an interlayer material without additional layers, and, subsequently, increase the overall resilience of the system.

**Figure 1:**
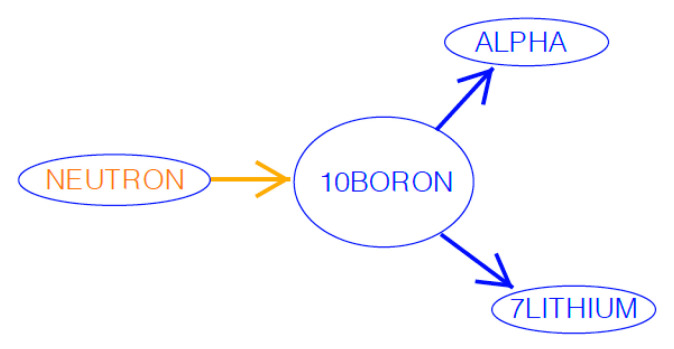
Nuclear bombardment of 10Boron with neutrons, emitting an Alpha particle and a 7Lithium particle.

The isotope 10Boron has a very high neutron capture cross-section of roughly 3840 barns for low energy thermal neutrons, making it a good candidate for capturing the neutron radiation [12]. Furthermore, the resultant products of the neutron capture, alpha emission (n, α) nuclear bombardment reaction are a sTable 7lithium isotope and an alpha (helium ion) emission. The alpha emission carries an energy of 1.78 MeV, and the 7lithium isotope recoils, conserving momentum, and carries an energy of 1.01 MeV [9]. These energetic particles are subsequently used in the crosslinking mechanism for the strengthening of polymers. A diagram showing this nuclear bombardment reaction is shown in Figure 1.

**Figure 2:**
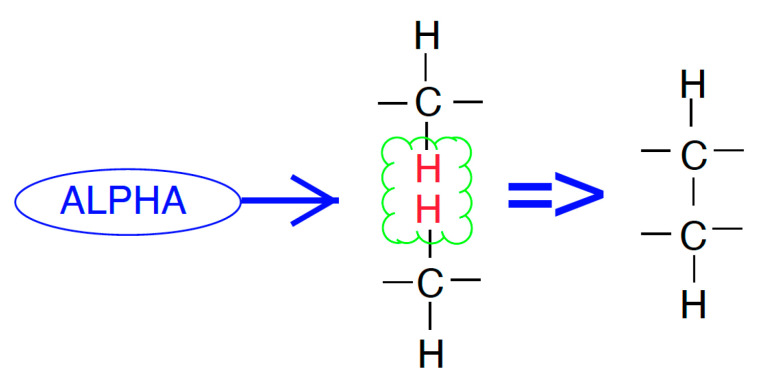
An alpha particle interacting with the hydrogen found in adjacent polyethylene chains; ideally creating a crosslink between the adjacent chains’ carbon elements.

Polymers are formed by chains of molecules which are largely composed of hydrogen and carbon. If adjacent polymer chains are exposed to high energy charged particles it is possible for the hydrogen to break free, leaving the carbon available to form new bonds and crosslink, increasing the overall resilience of the material. This could take place through different mechanisms such as electron stripping from the bonded elements, where the new state will seek out a suitable bond that brings the system to its lowest energy state. This is how crosslinking is achieved in the polymer material. An example of this crosslinking mechanism can be seen in Figure 2.

### 1.3. Polymer Interlayer Materials

The polymer interlayer material selected for testing included Polyvinyl Butyral (PVB) and Ethylene Vinyl Acetate (EVA). These products are widely used and readily available in the window manufacturing industry. Tested specimens were selected from the same manufactured batch of product, eliminating inconsistencies that may result during the manufacturing process.

**Figure 3:**
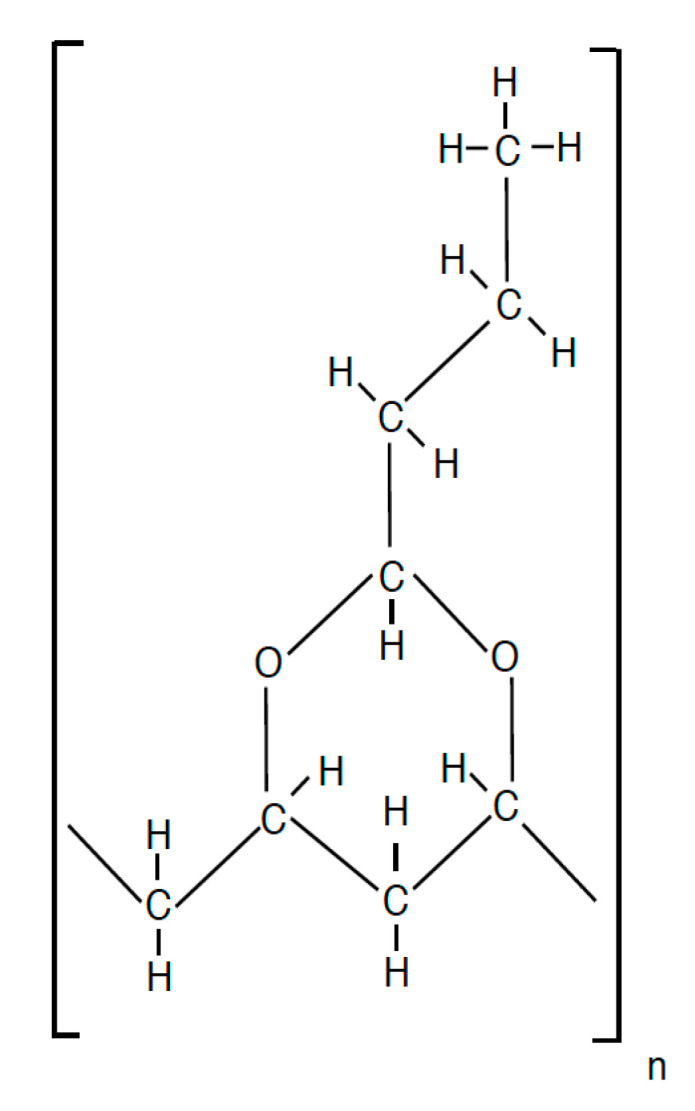
PVB chain structure.

PVB is a solid thermoplastic resin. It has been the standard laminated glass interlayer for the last 70 years. PVB is produced from the reaction of polyvinyl alcohol with butyraldehyde [13]. The chemical structure is identical for every manufacturer and is shown in Figure 3.

**Figure 4:**
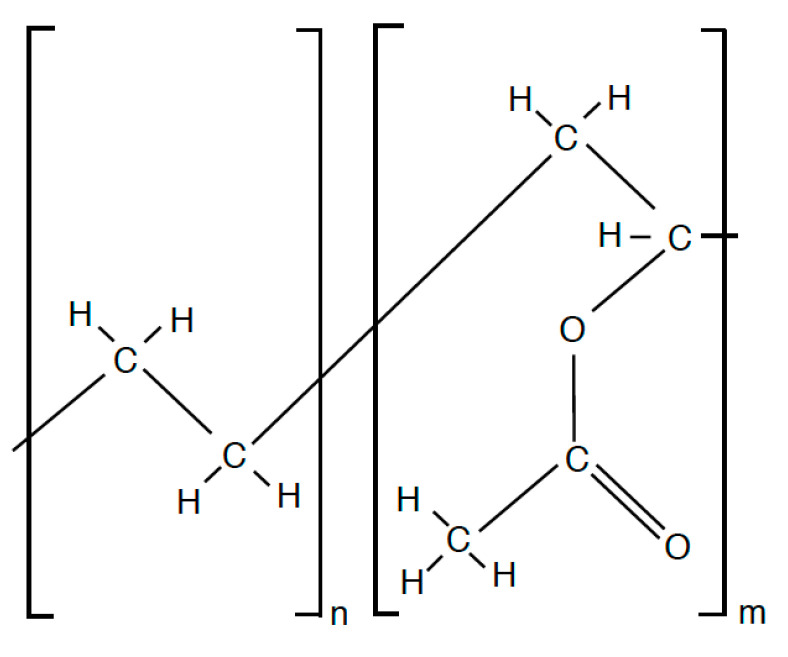
EVA Chemical Structure.

EVA is also a solid thermoplastic resin produced by the co-polymerization processing of ethylene (C_2_H_4_) and a vinyl acetate monomer (C_4_H_6_O_2_) in a high-pressure reactor. It is often used in architectural glass panels as well as solar panels. The copolymer chemical structure can be seen in Figure 4. Both PVB and EVA show promise in their chemical structure for susceptibility towards crosslinking through their plentiful hydrogen/carbon bonds.

## 2. Material Characterization Techniques

To determine if the crosslinking mechanism has been successful in the interlayer materials, several methods of material evaluation were reviewed to identify clear, repeatable changes in the mechanical properties. The material characterization techniques utilized in evaluating the performance changes of the interlayer material include different tensile loading methods and hardness evaluation methods [14].

Two tensile testing techniques were applied for the polymer interlayer material. The quasi-static tensile test meets ASTM D638-14 [15] test standards for material testing. It is a standard method of testing that pulls a specimen to failure at a low strain rate. This produces a stress/strain curve characteristic to the material. The high strain rate tensile test method is used to identify tensile loading characteristics that would be seen under blast conditions and is oftentimes referred to as a dynamic tensile test [16]. This method pulls the specimen to failure in a very short amount of time. This also produces a stress/strain curve characteristic to the material [17,18].

The technique used to evaluate hardness varied depending on the type of material, the thickness of material, and the conditions under which testing occurred. The types of hardness testing utilized with these interlayer materials included nano-indentation and scratch testing. Nano-indentation [19,20] was used to determine hardness and elasticity in the thin sheet polymer interlayer materials. Scratch testing was conducted on the material sheets to determine the resistance to scratching. Anton Paar instrumentation was used for these material evaluations.

## 3. Surface Treatment Techniques

Early treatment methods during this work were developed using polymeric material that had boron dispersed evenly throughout the material. This had advantages for bulk treatment at a significant depth within the material. For thinner materials such as polymer interlayers (0.381–0.762 mm or 0.015–0.030 inches thick), a surface treatment method was developed that could affect a significant depth of the material. The advantage of this technique is that the materials can be treated without the additional manufacturing step of boron doping that is required for bulk treatment.

A series of tests were performed on samples of commonly used interlayer materials that include EVA and PVB. These tests exposed samples of the material to a low flux rate of neutrons (8.4 × 10^8^ N/cm^2^/s). The neutron source was developed at the University of Missouri Research Reactor (MURR) and has been utilized for a variety of boron neutron capture research [21,22,23]. The irradiation set-up consisted of a layered system to gently compress the polymer interlayer material between a boron nitride plate and an aluminum plate. The aluminum neutron capture cross-section is low, making the aluminum plate nearly transparent to the neutrons as they pass through towards the boron rich plate. Minimal thermal neutron capture is expected within the polymer material as the neutrons continue toward the boron rich plate. Once the neutrons bombard the boron nitride plate, the high cross-section 10Boron will interact with the neutrons and emit energetic particles into the surface layer of the polymer interlayer. As naturally occurring boron contains approximately 19.9% of the isotope 10Boron, the neutron capture should produce significant quantities of energetic particles required to develop crosslinking.

Polymeric sheet material EVA with a thickness of 0.381 mm and PVB with a thickness of 0.762 mm were selected for the initial treatment. As outlined in [9], the boron treatment has a range of approximately 10 microns. This range, although limited, still has application on the thin interlayer material through surface treatment techniques, allowing treatment of nearly 3% of the 0.381 mm thick EVA.

To verify that the changes in the material behavior of the polymer interlayer are due to the boron surface treatment mechanism, samples of EVA and PVB were also irradiated without the boron nitride. The reason for pursuing this test was to rule out effects aside from the expected boron neutron capture. The absence of boron allows for the observation of any effects that could be caused by gamma radiation, beta radiation, or any other unknown factor during the treatment. Aluminum plates were used on each surface and irradiated in the same manner as the boron treated material. These samples were then tested with a quasi-static tensile test for comparison with the unirradiated control.

EVA and PVB were then irradiated with the boron nitride ceramic plate. The aluminum plate was used on the surface facing the beam. These irradiated samples were then tested with a quasi-static tensile test, high strain rate tensile test, nano-indentation test, and scratch test for comparison to the control.

Optimal neutron dose amounts were further investigated by the surface treatment method effect on the EVA interlayer material. Neutron flux was 8.4 × 10^8^ N/cm^2^/s and samples were irradiated for 0 h, 10 h, 20 h, 40 h, and 100 h. It was hoped that the results would show an optimal change in behavior. The material was evaluated with a quasi-static tensile test.

## 4. Material Characterization Results

Both interlayer materials, EVA and PVB, were irradiated initially for a 100-h period. Material characterization was conducted to determine changes in their properties following the treatment. A test matrix that outlines the various treatment methods and the evaluation method can be reviewed in Table 1.

### 4.1. Interlayer Material Irradiation without Boron

**Figure 5:**
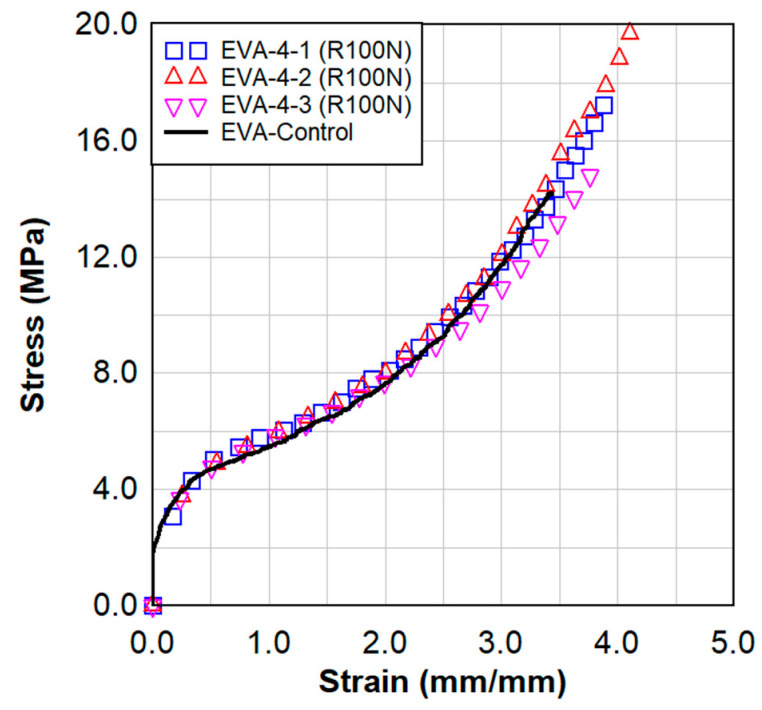
Static testing of EVA−4 (R100N) specimens, with aluminum-EVA-aluminum configuration, in comparison to the virgin EVA material.

As described in Section 3, the EVA material was irradiated without boron to evaluate the effects of radiation alone on the specimen. The results from static testing can be seen in Figure 5. Static testing was performed on three samples cut from the irradiated specimen EVA-008, and then compared to a control tested on the virgin EVA material. Reviewing the graphed results in Figure 5, the radiated sample without boron behaves very similar to the control EVA sample. This is a clear indication that any outside radiation effects have had very little impact on the material properties of the specimen.

### 4.2. Interlayer Material Irradiation with Boron

EVA and PVB samples were irradiated with boron to evaluate the effects of the surface treatment technique on the specimen. In this treatment the boron plate was included, as described Section 3.

**Figure 6:**
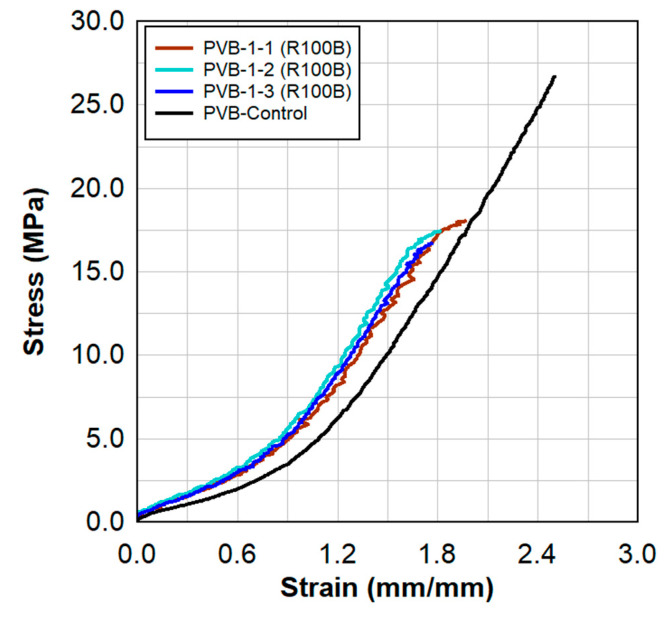
Static tensile test results for the PVB−1 specimens and a comparison to the virgin PVB material.

**Figure 7:**
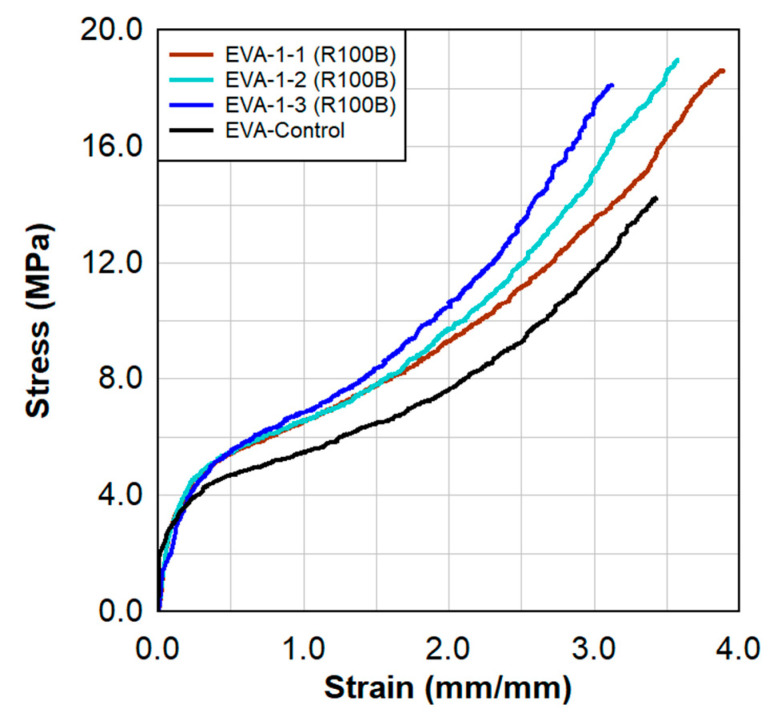
Static tensile test results for the EVA−1 specimens and a comparison to the virgin EVA material.

Static tensile test results are shown in Figure 6 and Figure 7. These results show a comparison between the virgin material that was not exposed to radiation and a series of samples tested following the previously described surface treatment technique. There were clear increases in the stress capacities at lower strains, indicating a stiffer material.

The increases in the stress capacity by 40% contribute to the overall increases in toughness by 35%. Toughness is indicative of the amount of energy absorbed during the tensile test and is quantified by the area under the stress—strain curve.

**Figure 8:**
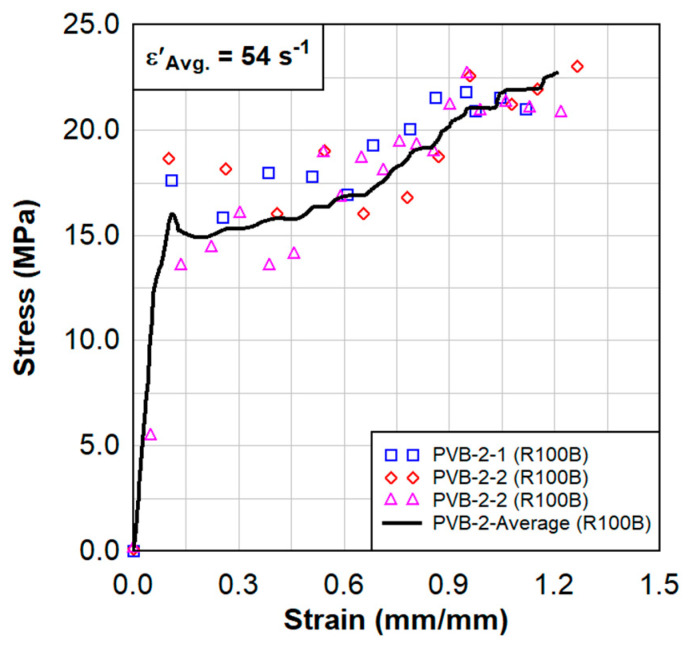
Dynamic tensile test results from three samples of radiated PVB−2 specimens.

**Figure 9:**
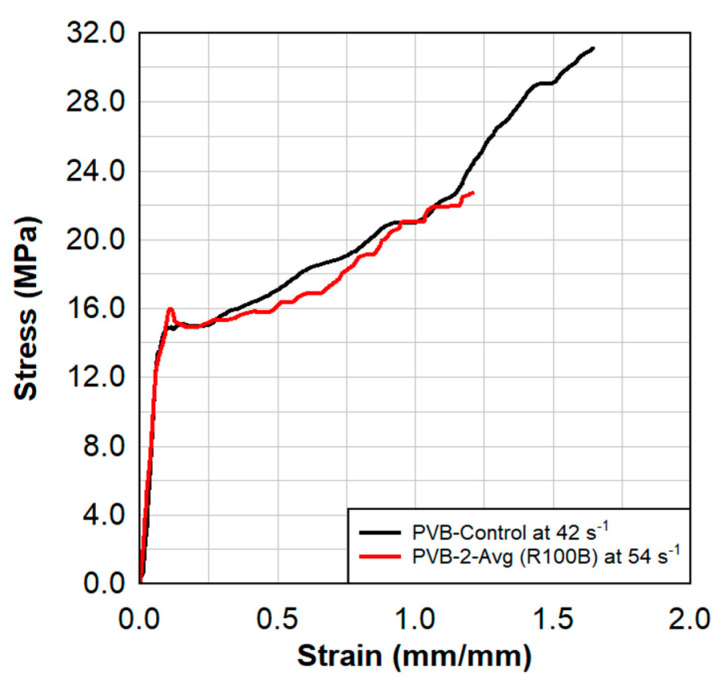
Dynamic tensile test results of the average of the three radiated PVB−2 specimens in comparison to the virgin PVB.

**Figure 10:**
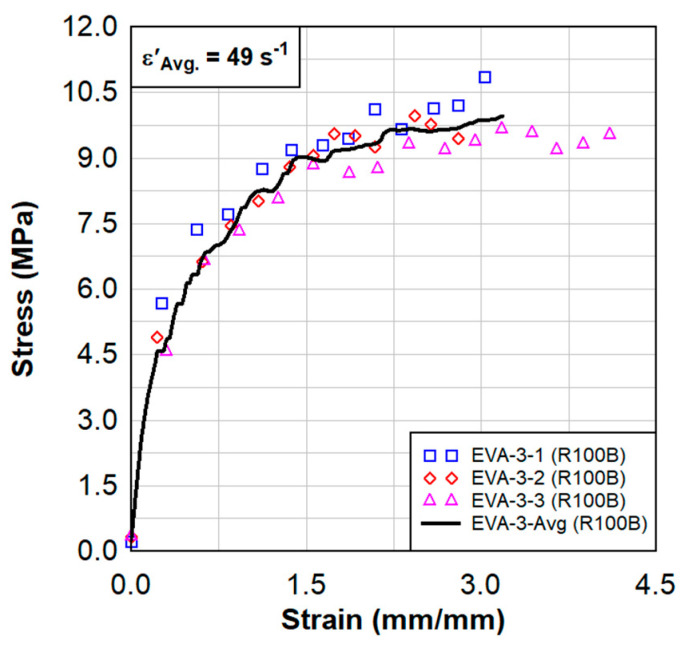
Dynamic tensile test results from three samples of radiated EVA−3 specimens.

**Figure 11:**
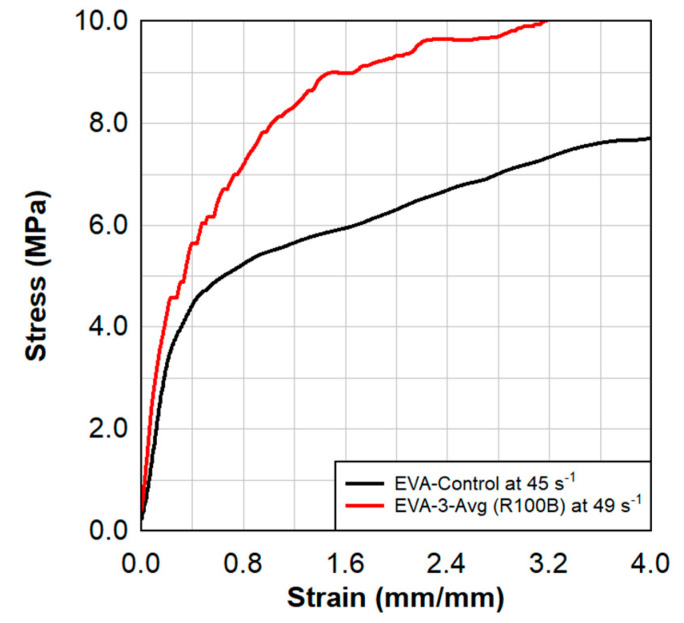
Dynamic tensile test results of the average of the radiated EVA−3 specimens in comparison to the virgin EVA.

**Figure 12:**
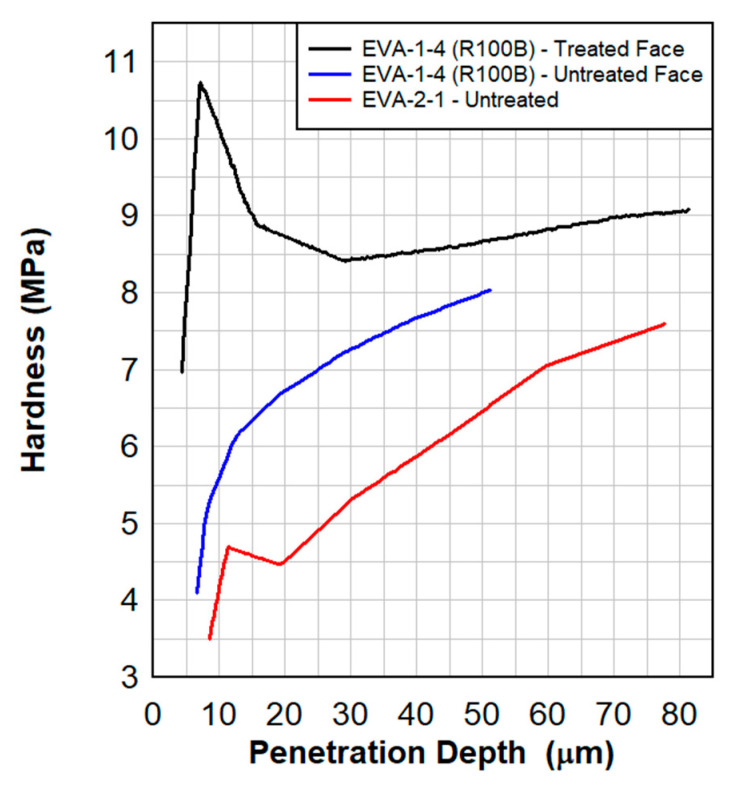
Hardness as a function of the penetration depth for the radiated and virgin EVA specimens. The treated material shows significant hardening in the first 10 µm of the surface, which could resist the cutting of polymer by glass shards during a blast.

**Figure 13:**
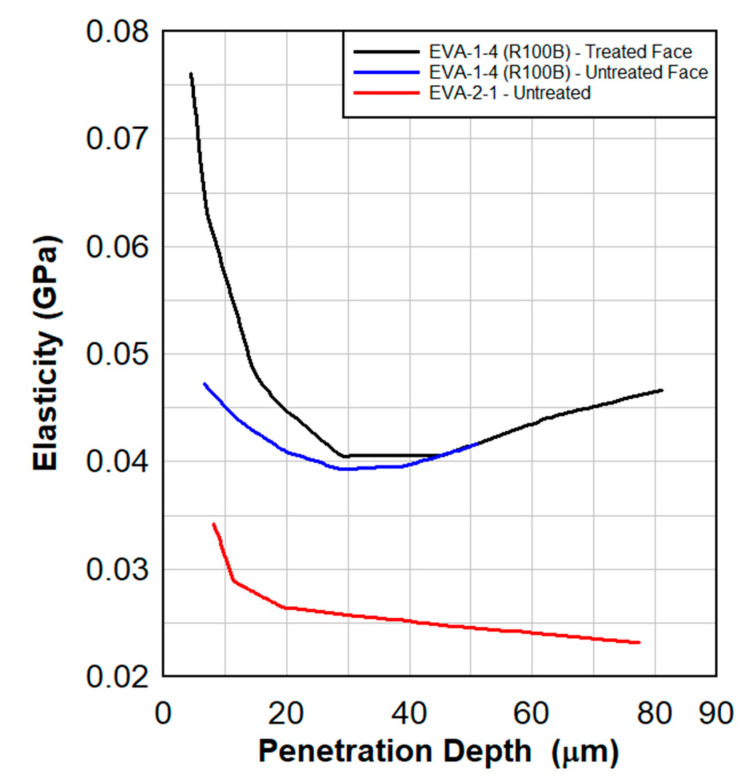
Elasticity as a function of the penetration depth for the radiated and virgin EVA specimens. Treated material shows a significant elasticity in the first 10 µm of the surface.

Dynamic tensile test results are shown in Figure 8, Figure 9, Figure 10 and Figure 11. These results also show a comparison between the virgin material that was not exposed to radiation and a series of samples tested following the previously described surface treatment technique. Although the PVB samples did not show significant material stiffening, the EVA continued to show signs of stiffening which might be attributed to the crosslinking occurrence from the treatment. As EVA continuously showed more pronounced results, subsequent treatments focused primarily on EVA. Indenter testing was performed by Anton Paar. Results can be seen in Figure 12 and Figure 13. A test matrix that indicates the specimen for indentation and scratch testing can be seen in Table 1.

In Figure 12 and Figure 13, in approximately the first 10 µm of the treated material, there is a 100% increase in the hardness and a 40% increase in the elasticity of the treated EVA. Note that the range of the crosslinking radiation is approximately 10 µm into the material. A hardened surface can resist the cutting of the polymer by glass shards during a blast.

**Figure 14:**
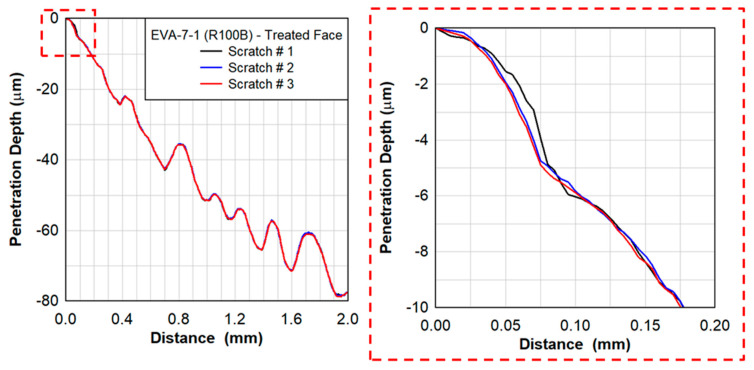
Scratch testing of EVA−7−1 showing the penetration depth as a function of the distance with a scratch rate of 4 mm/min.

**Figure 15:**
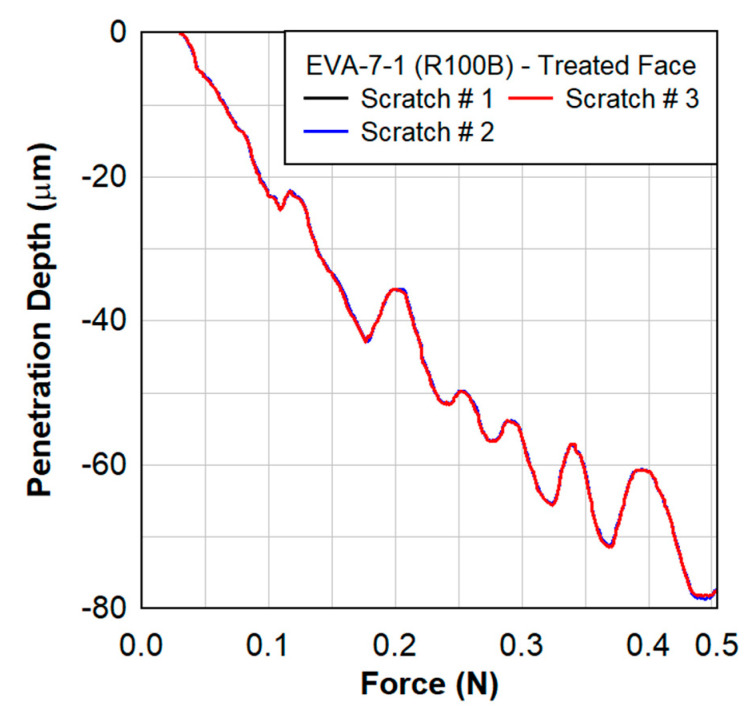
Scratch testing on EVA−07−1 showing the penetration depth as a function of the force applied with a scratch rate of 4 mm/min.

Scratch testing was performed by Anton Paar on the treated and untreated faces of the EVA specimens. Figure 14 shows the penetration depth as a function of the scratch distance at a rate of 4 mm/min for three different scratch tests on the sample EVA-014-001-R100B. The results shown in Figure 15 are for the treated face of the EVA specimen only. A close-up of the penetration depth of 10 µm is also shown in Figure 14. The applied force was increasing at a constant rate from 0.03 N to 0.50 N over a total distance of 2 mm. The penetration depth as a function of force is shown in Figure 15.

**Figure 16:**
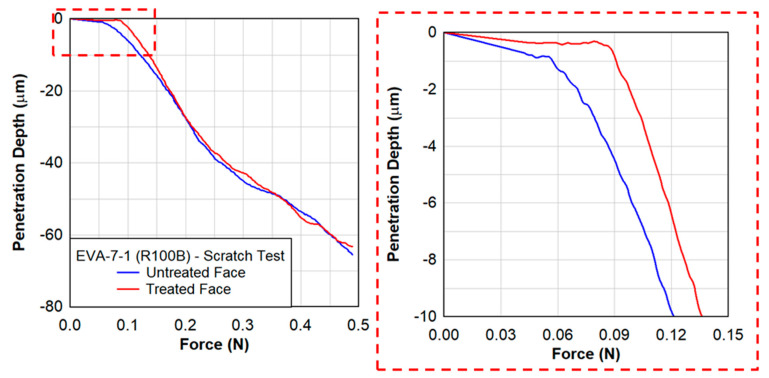
Scratch testing on EVA−7−1 showing the penetration depth as a function of the distance at a scratch rate of 85 mm/min.

**Figure 17:**
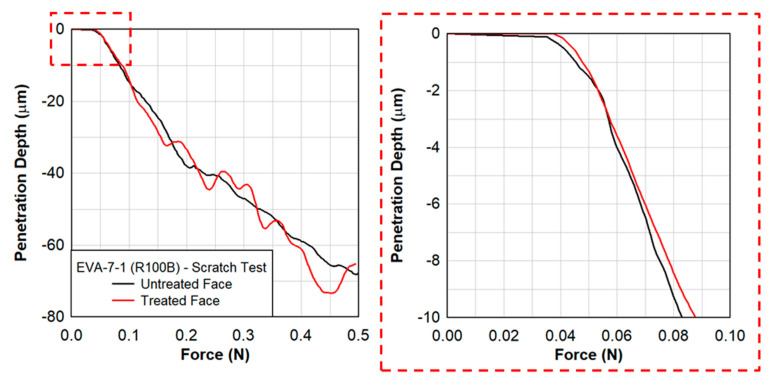
Scratch testing on EVA−7−1 showing the penetration depth as a function of force applied at a scratch rate of 20 mm/min.

The effect of the radiation treatment is shown in Figure 16, where the treated face produced a larger penetration resistance than the untreated face. Over the 10 µm depth of the treated face, the resistance was on average 30% larger than the untreated face. This was observed at a higher rate of scratch testing of 85 mm/min. However, at slow rate of scratch testing of 20 mm/min, the difference in the force resistance was not as noticeable (Figure 17).

Overall, the scratch testing provided supporting material characterization data indicating that the treated surface of the EVA material was improved to provide a higher resistance to being scratched. This is further indicative of a crosslinking mechanism taking place from the surface treatment process.

### 4.3. Dose Effects on Interlayer Material

**Figure 18:**
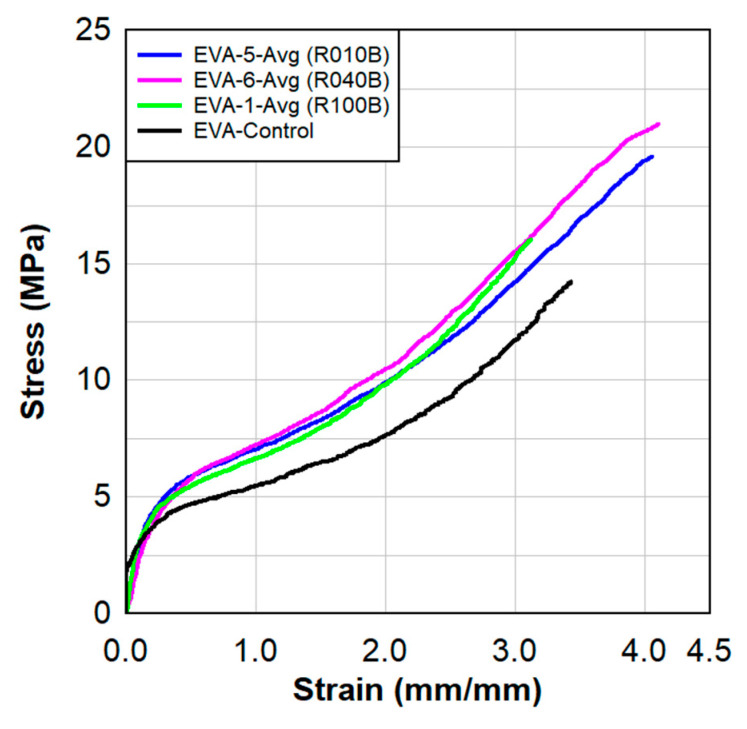
Radiation dose effect on the tensile strength of EVA.

Samples that were irradiated at the same 8.4 × 10^8^ N/cm^2^/s neutron flux for varying time durations were tested with the static tensile testing process for comparison. This evaluation will provide insight into the effects of progressively larger doses of neutron radiation. Samples included irradiation durations of 0-, 10-, 40-, and 100-h for comparison. Three individual test coupons were made for tensile evaluation regarding each irradiation, and the average of the three tensile tests are provided. Results from the study can be seen in Figure 18.

As seen in Figure 18, all radiated specimens exhibited a higher stiffness compared to the control specimen. There was a significant stiffness behavior change at even the lowest 10-h irradiation, specimen EVA-5. The 40-h irradiation, specimen EVA-6, showed a slightly higher stiffness than the 10-h irradiation. As the duration reached the 100-h length, it appears there may even have been some degradation. This may be indicative of damage due to overtreatment. It could be that the most effective treatment dose may be closer to the 40-h duration, and that there is still a high level of efficiency at only the 10-h dose.

## 5. Conclusions

Polymer treatment through the boron neutron capture radiation treatment has produced polymer interlayers with potential increased resilience towards blast threats. The research to date has evaluated treated polymers in accordance with theoretical research and shown that the hardening and increased elasticity of the material can be initiated through treatment, thus indicating crosslinking behavior. The research has developed methods beyond the radiation of boron doped polymers, through surface treatment, and applied them to polymer interlayers typically used in window systems. These results showed distinct changes in the material behavior, particularly with the EVA interlayer material, utilizing multiple material characterization methods to fully understand and verify the behavior change. The harder surface may assist in the cutting of the interlayer surface by glass shards; the overall toughness of the polymer material is increased allowing for a higher energy absorption, and an overall improvement of the window performance in blast conditions is expected. The treatment technique can further be applied to window interlayer products to determine optimal material characteristics for blast conditions.

## Figures and Tables

**Table 1:** Test matrix of specimen.

Specimen	Radiation Treatment	Evaluation Method
PVB-Control	No radiation, virgin material	Static and dynamic tensile
PVB-1-1	Radiated at low flux, 100 h, boron plate (R100B)	Static tensile
PVB-1-2	Radiated at low flux, 100 h, boron plate (R100B)	Static tensile
PVB-1-3	Radiated at low flux, 100 h, boron plate (R100B)	Static tensile
PVB-2-1	Radiated at low flux, 100 h, boron plate (R100B)	Dynamic tensile
PVB-2-2	Radiated at low flux, 100 h, boron plate (R100B)	Dynamic tensile
PVB-2-3	Radiated at low flux, 100 h, boron plate (R100B)	Dynamic tensile
EVA-Control	No radiation, virgin material	Static and dynamic tensile
EVA-1-1	Radiated at low flux, 100 h, boron plate (R100B)	Static tensile
EVA-1-2	Radiated at low flux, 100 h, boron plate (R100B)	Static tensile
EVA-1-3	Radiated at low flux, 100 h, boron plate (R100B)	Static tensile
EVA-1-4	Radiated at low flux, 100 h, boron plate (R100B)	Indentation
EVA-2-1	No radiation, virgin material	Indentation
EVA-3-1	Radiated at low flux, 100 h, boron plate (R100B)	Dynamic tensile
EVA-3-2	Radiated at low flux, 100 h, boron plate (R100B)	Dynamic tensile
EVA-3-3	Radiated at low flux, 100 h, boron plate (R100B)	Dynamic tensile
EVA-4-1	Radiated at low flux, 100 h, no boron (R100N)	Static tensile
EVA-4-2	Radiated at low flux, 100 h, no boron (R100N)	Static tensile
EVA-4-3	Radiated at low flux, 100 h, no boron (R100N)	Static tensile
EVA-5-1	Radiated at low flux, 10 h, boron plate (R010B)	Static tensile
EVA-5-2	Radiated at low flux, 10 h, boron plate (R010B)	Static tensile
EVA-5-3	Radiated at low flux, 10 h, boron plate (R010B)	Static tensile
EVA-6-1	Radiated at low flux, 40 h, boron plate (R040B)	Static tensile
EVA-6-2	Radiated at low flux, 40 h, boron plate (R040B)	Static tensile
EVA-6-3	Radiated at low flux, 40 h, boron plate (R040B)	Static tensile
EVA-7-1	Radiated at low flux, 100 h, boron plate (R100B)	Scratch

## Data Availability

Not applicable.
